# Establishing the Approach of Norm Balance toward Intention Prediction across Six Behaviors under the Theory of Planned Behavior

**DOI:** 10.3390/pharmacy11020067

**Published:** 2023-03-31

**Authors:** Yifei Liu, Karen B. Farris, Dhananjay Nayakankuppam, William R. Doucette

**Affiliations:** 1 Division of Pharmacy Practice and Administration, University of Missouri–Kansas City School of Pharmacy, Kansas City, MO 64108, USA; 2 Department of Clinical Pharmacy, College of Pharmacy, University of Michigan, Ann Arbor, MI 48109, USA; 3 Department of Marketing, Tippie College of Business, University of Iowa, Iowa City, IA 52242, USA; 4 Department of Pharmacy Practice and Science, College of Pharmacy, University of Iowa, Iowa City, IA 52242, USA

**Keywords:** The Theory of Planned Behavior, intention, subjective norm, self-identify, Norm Balance

## Abstract

*Background*: An innovative approach of Norm Balance is proposed under the Theory of Planned Behavior (TPB). In this approach, the measurement score of subjective norm is weighted by the relative importance of others, and the measurement score of self-identity is weighted by the relative importance of self. The study objective was to examine the effect of Norm Balance to predict behavioral intentions in two groups of college students. *Methods*: Cross-sectional surveys were used in two studies. For 153 business undergraduates, Study 1 examined three common intentions: eating a low-fat diet, exercising regularly, and dressing business-like. For 176 PharmD students, Study 2 examined three pharmacy-related intentions: informing relatives about counterfeit medications, buying prescription medications online, and completing a pharmacy residency. The relative importance of others vs. self was measured by asking study subjects to allocate 10 points between important others and oneself. Two sets of regressions were conducted and compared across six intentions using the traditional model and the Norm Balance model. *Results*: The 12 regressions explained 59–77% of intention variance. The variance explained by the two models was similar. When subjective norm or self-identity was non-significant in the traditional model, the corresponding Norm Balance component was significant in the Norm Balance model, except for eating a low-fat diet. When both subjective norm and self-identity were significant in the traditional model, the two Norm Balance components were significant in the Norm Balance model with increased coefficients. *Conclusions*: The proposed approach of Norm Balance provides a different view about the significance and coefficients of subjective norm and self-identity toward intention prediction.

## 1. Introduction

### 1.1. Background

The Theory of Planned Behavior (TPB) is an essential framework to examine behaviors. According to the TPB, the principal determinant of behavior is behavioral intention, and behavioral intention is determined by attitude toward behavior, subjective norm, and perceived behavioral control [1,2]. However, the current TPB constructs may not be sufficient causes of intention or behavior, suggesting the addition of other variables to explain variance in intention or behavior [2,3]. Self-identity is a concept from identity theory and reflects the extent to which individuals perceive themselves as taking on a particular role in society [4,5]. The more a person perceives oneself as performing a particular role, the more self-identity will affect behavioral intention [6]. Self-identity has been shown to be a significant predictor of intention in the TPB for a variety of behaviors [6,7,8,9,10,11,12,13,14,15,16,17,18,19]. 

Subjective norm is a normative concept—the assessment of social pressures placed on an individual to perform a behavior [20]. There are two ways to measure the TPB constructs including subjective norm—indirect measures and direct measures [21]. Using indirect measures, a person’s subjective norm is represented by normative beliefs weighted by the motivation to comply with the referent [20]. Using direct measures, subjective norm is determined by single items, such as “most people important to me think I should perform the behavior” [6,12,13,21]. Direct measures of the TPB constructs appear to be more strongly associated with intention than indirect measures and have been used in studies incorporating self-identity into the TPB [6,7,8,10,12,13,14,16,21].

Self-identity is also a normative construct which influences intention [6,22], but it differs from subjective norm. Self-identity is usually measured by single items, such as “I think of myself as someone as a behavior performer” [6,12,13]. Self-identity represents the social context on an actor, namely the category of an actor with identifiable social characteristics [7]. In contrast, subjective norm represents the pressure from an actor’s reference groups on the behavioral performance [20]. In other words, the focus of self-identity is on oneself (personal norm), “whether I think I should perform a behavior”, but the emphasis of subjective norm is one’s reference group (referent norm), “whether my important others think I should perform a behavior”.

### 1.2. Theorization

**Figure 1:**
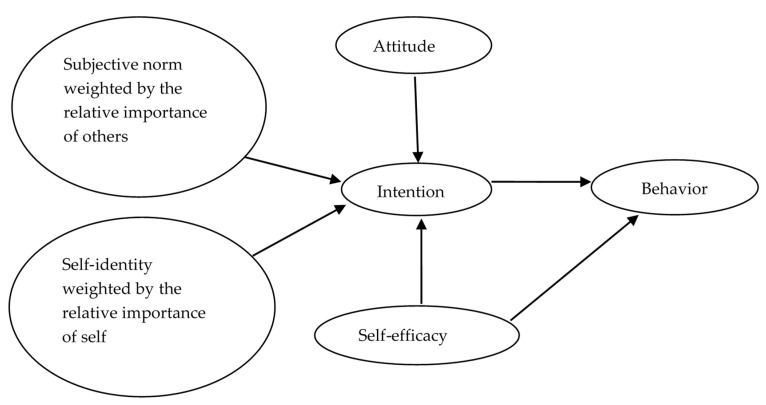
The proposed approach of Norm Balance under the TPB.

We proposed an innovative approach of Norm Balance in which self-identity and subjective norm need two parameters to be fully measured. Norm Balance is a theoretical approach rather than a construct. In this approach, subjective norm and self-identity are theorized under an overall domain of “norm”, and each has a strength and proportion within the domain of “norm”. The strength is represented by the measurement scores of subjective norm and self-identity. The proportion indicates the share each construct occupies in the domain of “norm”, which is represented by the relative importance of others vs. self. Under this approach, for an individual, the measurement score of subjective norm should be weighted by the relative importance of others, and the measurement score of self-identity should be weighted be the relative importance of the self (Figure 1). For instance, one’s important others think the individual should perform a behavior but the individual does not think so. If the person regards oneself as a more important source impacting the behavioral decision, the person may not intend to perform the behavior. To the contrary, if the person considers important others to be a more influential source impacting the behavioral decision, the person may intend to perform the behavior.

In addition, Ajzen theorized a hierarchical model of perceived behavioral control where perceived behavioral control is represented by two subcomponents: self-efficacy and controllability [23]. Self-efficacy has been consistently shown to be a stronger predictor of both intention and behavior than controllability [6,12,13,24]. Therefore, we used self-efficacy to represent perceived behavioral control. 

### 1.3. Objective

The objective of this research was to examine the effect of Norm Balance to predict behavioral intentions in two groups of college students. If Norm Balance is a valid approach for intention prediction, the TPB could be modified to improve its predictive utility regarding intention. This research only examined intention prediction in Figure 1.

## 2. Methods

### 2.1. Design and Samples

Cross-sectional surveys were employed in two studies using convenience samples. Inclusion criteria of Study 1 were 153 undergraduates who took a course at a college of business. Inclusion criteria of Study 2 were 102 second-year PharmD students who took a course, and 74 third-year PharmD students who took one of two courses at a college of pharmacy. Hard copies of the instrument (Appendix A) were distributed to the students and collected from them while they were in class. There was no incentive to fill out the survey and the participation was voluntary. 

In a regression analysis, as a rule-of-thumb, the sample size should be equal to or greater than “50 + 8 × m for the multiple correlation”, or “104 + m for the partial correlation”, where “m” is the number of predictors [25]. We expected to have nine independent variables in a regression model, so we needed a minimum of 50 + 8 × 9 = 122 subjects in either study. This estimated sample size of 122 was also sufficient (>104 + 9 = 113) to test individual predictors. 

Study 1 examined three common behavior intentions: (1) eating a low-fat diet during the next week; (2) exercising for at least 20 min, three times during the next week; and (3) dressing in a business manner in school during the next week. Study 2 examined three pharmacy-related behavioral intentions: (1) informing relatives about the risk of counterfeit medications when they consider buying prescription medications on the internet; (2) buying prescription medications on the internet when having a prescription; and (3) completing a pharmacy practice residency in the future. The robustness of Norm Balance could be demonstrated by choosing different behavioral intentions in the two studies.

The number of intentions were limited to three for each study to reduce the response burden on participants. In Study 1, eating a low-fat diet and regular exercising are behaviors well examined under the TPB with addition of self-identity [9,11,12,13,16,17]. Then the social situations of the other intentions were altered, since Norm Balance may vary across different social contexts. For example, dressing in a business manner in school (Study 1) is relatively public, while buying prescription medications online (Study 2) is relatively private, though the two study samples were different. In addition, informing relatives about the counterfeit risk of prescription medications purchased online (Study 2) is in a family setting, while completing a pharmacy practice residency (Study 2) is in a professional setting. Furthermore, the intentions in Study 1 were for the shorter term, whereas the intentions in Study 2 were for the longer term. 

### 2.2. Measures 

Both studies assessed attitude toward behavior, subjective norm, self-efficacy, self-identity, and intention for each behavior. They also assessed the relative importance of others vs. self and control variables. Measures of attitude toward behavior, subjective norm, self-efficacy, self-identity, and intention were included in Appendix A. They were derived from reliable and valid multi-item instruments with 7-point response scales from the studies of Armitage and Connor by changing the context of the behavior [12,13]. The phrase “If I wanted to” was added to self-efficacy measures according to the suggestion of Rhodes and Courneya to assume motivation and to “reduce inadvertent tautological theorizing” [26]. 

The relative importance of others vs. self was measured by the following item:

“Please allocate 10 points between the two sources below to indicate the extent of their impact on your decision to… Please use whole numbers.

People who are important to you____; Yourself____”.

Control variables included age, gender, race, marital status, and whether having children. Based on the openness of the TPB to include additional variables, control variables were used to account for intention variance due to differences in demographic and other characteristics [2].

### 2.3. Data Analyses

For each behavior, overall measures of attitude toward behavior, subjective norm, self-efficacy, self-identity, and intention were calculated for each person by taking the mean of the corresponding items. The frequency, means, standard deviations, and bivariate correlations for the measured variables were calculated. Reliability analyses were performed for the multiple-item measurements. We hypothesized that the approach of Norm Balance would explain intention differently than simply using the measurement scores of subjective norm and self-identity. The following two multiple regression models across six behavioral intentions were conducted and compared. Both studies were approved by the Institutional Review Board of the University of Iowa. 

The traditional model: Intention was regressed on attitude, subjective norm (measurement score), self-efficacy, self-identity (measurement score), and control variables. 

The Norm Balance model: Intention was regressed on attitude, the relative importance of others × measurement score of subjective norm, self-efficacy, the relative importance of self × measurement score of self-identity, and control variables. 

## 3. Results

The response rate of Study 1 was 98% (150 students out of 153), and the response rate of Study 2 was 93% (157 students out of 169). For both studies, the average respondent was in their 20s, white, single, and had no children (Table 1). The variables had high reliability (Cronbach’s alpha > 0.7), except for subjective norm (measurement score) for informing relatives about the risk of counterfeit drugs, and self-identity (measurement score) for informing relatives about the risk of counterfeit drugs and buying prescription drugs online (Table 2). 

Across five intentions, the average relative importance of others vs. self was about 3 vs. 7, given 10 points to allocate; for the intention to inform relatives about counterfeit medications, the average relative importance of others vs. self was 4.5 vs. 5.5 (Table 3). In general, the subjects perceived themselves as having more influence than their important others on behavioral decisions (Table 3 and Table 4). For each intention, there were subjects who did not care at all about the opinions of their important others (Table 4). For example, 19.5% of the PharmD students did not believe that their important others would impact their behavioral decision to purchase prescription drugs online. Additionally, the relative importance of others or self had low and non-significant correlations with the TPB constructs, which ranged from −0.27 to 0.27. 

The regressions using the traditional model accounted for 60–77% of intention variance (Table 5). The regressions of Norm Balance model explained 59–77% of intention variance (Table 6). The variance explained by the two models was similar (difference ≤ 1%), except for the intention of purchasing prescription drugs online (the Norm Balance model explained 3% more). When subjective norm or self-identity was non-significant in the traditional model, the corresponding Norm Balance component was significant in the Norm Balance model, except for the intention of eating a low-fat diet (Table 7). When both subjective norm and self-identity were significant in the traditional model, two Norm Balance components were significant in the Norm Balance model with increased coefficients.

## 4. Discussion

### 4.1. Predictive Utility of the Approach of Norm Balance 

The regression results indicate that the Norm Balance components have a similar overall effect as using unweighted measurement scores of subjective norm and self-identity to explain intention variance. However, the proposed approach of Norm Balance provides a different view about the significance or coefficients of subjective norm and self-identity, because there were clear changes from the traditional model (Table 7). For example, for the intention to complete a pharmacy residency in Study 2, the traditional model showed that subjective norm (measurement score) was the second strongest intention predictor, while self-identity (measurement score) was non-significant. However, after considering the relative importance, self-identity became the second strongest intention predictor. That is, with more variation added to measurement scores using the relative importance of others vs. self, self-identity became a more powerful predictor for an intention than subjective norm. Another example is the intention to buy prescription medications on the internet in Study 2. After considering the relative importance, subjective norm changed from a non-significant predictor to a significant predictor. The low and non-significant correlations between the relative importance of others vs. self and intention indicate that the relative importance does not independently explain intention. This is consistent with the theorization of the relative importance, namely using it to weigh measurement scores rather than using it as an intention predictor. The low correlations between the relative importance and the measurement score of subjective norm or self-identity suggest that the relative importance is not another way to measure subjective norm or self-identity. 

### 4.2. The Relative Importance of Others vs. Self

An interesting finding was that the average relative importance of others vs. self was around 3 vs. 7 (given 10 points to allocate) across five intentions (Table 3). In other words, the subjects tended to regard themselves as being over two times more important than their important others for most behavioral decisions. Even for a behavior which may be related to important others (e.g., informing relatives about counterfeit medications), the subjects still valued their own opinions more. Cultural variation in the self-concept also supports the existence of the relative importance of others vs. self in the proposed approach of Norm Balance. Western culture tends to be independent, where behaviors are associated with personal values; whereas eastern culture is more interdependent, where behaviors are made meaningful when using others’ values for reference [27]. “Independence vs. interdependence” is also the basis for other differences between western and eastern cultures, such as opinions toward achievement, self-criticism, equality, and so on [28]. The framework of individualism vs. collectivism provides an alternate view about cultural variation in the self-concept. For example, individualists are independent, prioritize their personal goals over collective goals, base their behaviors on attitudes and hedonism, and calculate the profit and cost of relationships [29]. Collectivists, on the other hand, are interdependent, subdue their personal goals in favor of collective goals, base their behaviors on norms and duties, and consider others’ needs in social exchange. Individualism is common in Western Europe and North America, whereas collectivism is common in Asia and Africa [30]. Not measuring the relative importance of others vs. self may lead to the misinterpretation of results or ineffective interventions. 

### 4.3. Limitations

There were three limitations in the studies. First, the reliability of self-identity (measurement score) for buying prescription drugs online was low (Cronbach’s alpha = 0.514). The three self-identity items (measurement score) were: (1) “I think of myself as an internet shopper of prescription medication”; (2) “I think of myself as someone who is concerned with getting prescription medication on the internet for a low price”; and (3) “I think of myself as someone who is concerned with the convenience of internet purchase of prescription medication”. These items may measure the different societal roles associated with internet shopping. For example, item (1) measured the role of “internet shopper”, but item (2) might measure the role of “price grabber”. Thus, the reliability was low. Second, the study subjects were college students with homogeneous demographics, such as age, education, ethnicity, and marital status. Therefore, the results may not be generalizable to other populations. Third, although the approach of Norm Balance accounted for 59–77% of the intention variance (Table 6), compared with the traditional model, it did not further improve the variance explanation. However, no improvement in explaining intention variance does not necessarily mean that this approach is invalid. It does offer another perspective to examine the significance or coefficients of subjective norm or self-identify (Table 7). 

### 4.4. Future Research

Three directions exist for further efforts exploring the approach of Norm Balance. First, the development of other measures to capture the relative importance of others vs. self. Second, the use of this approach to assess the same behavior intention for oneself vs. others, or across different cultures, and then a comparison of the results. For instance, how parents’ intention regarding the same health behavior varies for themselves vs. their children. Third, an examination of factors affecting this approach. According to the study results, it seems that behavior type or timeframe may have some influence.

## 5. Conclusions

In conclusion, the proposed approach of Norm Balance under the TPB explained similar intention variance to a traditional TPB model. However, it provides a different view about the significance or coefficients of subjective norm and self-identity.

## Figures and Tables

**Table 1:** Characteristics of the study subjects.

Variable	Business Undergraduates	PharmD Students
Age, years		
Mean (SD)	21.3 (1.9)	23.9 (2.8)
Range	18–33	21–36
Total N	150	152
Gender, no. (%)		
Female	59 (39.3)	110 (71.4)
Male	91 (60.7)	44 (28.6)
Total N	150 (100)	154 (100)
Ethnicity, no. (%)		
White	136 (90.7)	138 (90.2)
Non-White	14 (9.3)	15 (9.8)
Total N	150 (100)	153 (100)
Marital status, no. (%)		
Single (never married)	145 (96.7)	118 (76.8)
Non-single	5 (3.3)	36 (23.4)
Total N	150 (100)	154 (100)
Having children, no. (%)		
Yes	4 (2.7)	10 (6.5)
No	146 (97.3)	144 (93.5)
Total N	150 (100)	154 (100)

Note: N varies for the PharmD students due to missing values.

**Table 2:** Reliability Analyses of Multiple-item Construct Measurements.

Construct Variable	Business Undergraduates (Cronbach’ α)			PharmD Students (Cronbach’ α)		
	Eating a Low-Fat Diet	Exercising Regularly	Dressing Business-Like	Informing Relatives	Buying Rx Drugs	Completing Residency
Intention (3-item)	0.942 (n = 150)	0.912 (n = 150)	0.912 (n = 150)	0.875 (n = 155)	0.944 (n = 157)	0.972 (n = 155)
Attitude toward behavior (3-item)	0.799 (n = 150)	0.720 (n = 150)	0.829 (n = 150)	0.732 (n = 152)	0.884 (n = 152)	0.923 (n = 154)
Subjective norm (3-item, measurement score)	0.806 (n = 150)	0.800 (n = 149)	0.754 (n = 149)	0.680 (n = 153)	0.843 (n = 157)	0.802 (n = 151)
Self-efficacy (3-item)	0.850 (n = 150)	0.887 (n = 150)	0.877 (n = 150)	0.890 (n = 154)	0.845 (n = 154)	0.795 (n = 155)
Self-identity (3-item, measurement score)	0.883 (n = 150)	0.861 (n = 150)	0.884 (n = 150)	0.681 (n = 155)	0.514 (n = 157)	0.884 (n = 155)

Note: (1) N varies for the PharmD students due to missing values; (2) Values are in bold when Cronbach’ α < 0.7.

**Table 3:** Means and Standard Deviations of Construct Variables.

Construct Variable	Business Undergraduates Means (Std)			PharmD Students Means (Std)		
	Eating a Low-Fat Diet	Exercising Regularly	Dressing Business-Like	Informing Relatives	Buying Rx Drugs	Completing Residency
Intention (3-item) ^a^	4.28 (1.69) (n = 150)	5.96 (1.29) (n = 150)	2.95 (1.58) (n = 150)	6.08 (0.99) (n = 155)	1.31 (0.95) (n = 157)	4.23 (1.83) (n = 155)
Attitude toward behavior (3-item) ^a^	4.88 (1.26) (n = 150)	6.20 (1.00) (n = 150)	4.19 (1.36) (n = 150)	5.59 (1.03) (n = 152)	1.61 (1.10) (n = 152)	5.18 (1.61) (n = 154)
Subjective norm (3-item, measurement score) ^a^	4.72 (1.33) (n = 150)	5.70 (1.08) (n = 149)	3.94 (1.17) (n = 149)	5.86 (0.96) (n = 153)	1.83 (1.07) (n = 157)	4.73 (1.31) (n = 151)
Self-efficacy (3-item) ^a^	5.44 (1.26) (n = 150)	6.31 (1.08) (n = 150)	5.80 (1.32) (n = 150)	5.61 (1.24) (n = 154)	4.50 (2.04) (n = 154)	6.17 (1.01) (n = 155)
Self-identity (3-item, measurement score) ^a^	4.73 (1.36) (n = 150)	5.39 (1.36) (n = 150)	3.76 (1.42) (n = 150)	5.59 (1.10) (n = 155)	1.97 (1.21) (n = 157)	5.97 (1.07) (n = 155)
Relative importance of others ^b^	3.19 (1.63) (n = 150)	2.80 (1.50) (n = 150)	3.65 (1.99) (n = 150)	4.53 (2.07) (n = 146)	3.11 (2.20) (n = 149)	3.10 (1.92) (n = 149)
Relative importance of self ^b^	6.81 (1.63) (n = 150)	7.20 (1.50) (n = 150)	6.35 (1.99) (n = 150)	5.47 (2.07) (n = 146)	6.89 (2.20) (n = 149)	6.90 (1.92) (n = 149)

Note: (1) N varies for the PharmD students due to missing values. (2) ^a^ 1–7 scale, and ^b^ 10 points to allocate.

**Table 4:** Frequency of Relative Importance of Others vs. Self.

Frequency (%)	Business Undergraduates			PharmD Students		
Relative Importance of Others vs. Self	Eating Low-Fat Diet	Exercising Regularly	Dressing Business-Like	Informing Relatives	Buying Rx Drugs	Completing Residency
0/10	4 (2.7)	6 (4.0)	7 (4.7)	6 (4.1)	29 (19.5)	9 (6.0)
1/9	15 (10.0)	22 (14.7)	19 (12.7)	2 (1.3)	14 (9.4)	30 (20.1)
2/8	36 (24.0)	43 (28.7)	22 (14.7)	18 (12.3)	22 (14.8)	28 (18.8)
3/7	40 (26.7)	33 (22.0)	22 (14.7)	22 (15.1)	13 (8.7)	19 (12.8)
4/6	25 (16.7)	26 (17.3)	21 (14.0)	18 (12.3)	13 (8.7)	15 (10.1)
5/5	15 (10.0)	14 (9.3)	36 (24.0)	38 (26.0)	42 (28.2)	36 (24.2)
6/4	9 (6.0)	3 (2.0)	10 (6.7)	11 (7.5)	8 (5.4)	5 (3.4)
7/3	5 (3.3)	3 (2.0)	10 (6.7)	20 (13.7)	8 (5.4)	7 (4.7)
8/2	1 (0.7)	0	3 (2.0)	9 (6.2)	0	0
9/1	0	0	0	2 (1.3)	0	0
10/0	0	0	0	0	0	0
Total N	150 (100)	150 (100)	150 (100)	146 (100)	149 (100)	149 (100)

Note: N varies for PharmD students due to missing values.

**Table 5:** Prediction of Intention Using TPB with Addition of Self-identity (the Traditional Model).

Dependent Variable	Business Undergraduates			PharmD Students		
Intention	Eating a Low-Fat Diet (n = 150)	Exercising Regularly (n = 149)	Dressing Business-Like (n = 149)	Informing Relatives (n = 146)	Buying Rx Drugs (n = 146)	Completing Residency (n = 146)
Regression model						
Adjusted R square	0.65	0.77	0.60	0.66	0.68	0.68
Df	9	9	9	9	9	9
F	31.19 **	56.46 **	25.67 **	32.81 **	35.25 **	36.21 **
Standardized Beta						
Construct measurements						
Attitude toward behavior	0.64 **	0.25 **	0.33 **	0.26 **	0.78 **	0.67 **
Subjective norm (measurement score)	0.04	0.09 *	0.11	0.25 **	−0.01	0.19 **
Self-efficacy	0.05	0.36 **	−0.06	0.19 **	−0.09	0.00
Self-identity (measurement score)	0.13 *	0.36 **	0.40 **	0.32 **	0.17 **	0.08
Control variables						
Age	0.05	−0.02	−0.10	0.06	−0.04	0.00
Gender (male)	−0.19 **	−0.15 **	0.06	0.01	0.05	−0.01
Ethnicity (White)	0.05	0.03	−0.05	0.05	0.03	−0.01
Marital status (single)	−0.03	0.00	−0.14 *	0.09	0.02	−0.04
Having children	−0.06	0.04	0.08	−0.05	−0.07	−0.02

** significant at 0.01; * significant at 0.05. Note: The number of cases varied due to missing data.

**Table 6:** Prediction of Intention Using Norm Balance (the Norm Balance Model).

Dependent Variable	Business Undergraduates			PharmD Students		
Intention	Eating a Low-Fat Diet (n = 150)	Exercising Regularly (n = 149)	Dressing Business-Like (n = 149)	Informing Relatives (n = 141)	Buying Rx Drugs (n = 142)	Completing Residency (n = 141)
Regression model						
Adjusted R square	0.64	0.77	0.59	0.67	0.71	0.68
Df	9	9	9	9	9	9
F	30.74 **	54.92 **	24.62 **	33.17 **	39.92 **	33.83 **
Standardized Beta						
Construct measurements						
Attitude toward behavior	0.67 **	0.26 **	0.35 **	0.31 **	0.75 **	0.75 **
Relative importance of others × measurement score of subjective norm	0.03	0.27 **	0.27 **	0.66 **	0.14 **	0.15 *
Self-efficacy	0.06	0.38 **	−0.06	0.21 **	−0.09	−0.03
Relative importance of self × measurement score of self-identity	0.11	0.47 **	0.42 **	0.70 **	0.15 **	0.22 **
Control variables						
Age	0.04	−0.02	−0.11	0.06	−0.02	−0.03
Gender (male)	−0.19 **	−0.16 **	0.06	0.03	−0.01	−0.01
Ethnicity (White)	0.06	0.04	−0.06	0.05	0.02	−0.03
Marital status (single)	−0.04	−0.01	−0.14 *	0.06	0.04	−0.02
Having children	−0.05	0.03	0.08	−0.05	−0.08	0.01

** significant at 0.01; * significant at 0.05. Note: The number of cases varied due to missing data.

**Table 7:** Comparison of Regression Coefficients of Subjective Norm and Self-identity between Two Models.

Dependent Variable	Business Undergraduates			PharmD Students		
Intention	Eating a Low-Fat Diet (n = 150)	Exercising Regularly (n = 149)	Dressing Business-Like (n = 149)	Informing Relatives (n = 146)	Buying Rx Drugs (n = 146)	Completing Residency (n = 146)
Standardized Beta of the traditional model						
Subjective norm (measurement score)	0.04	0.09 *	0.11	0.25 **	−0.01	0.19 **
Self-identity (measurement score)	0.13 *	0.36 **	0.40 **	0.32 **	0.17 **	0.08
Standardized Beta of the Norm Balance model						
Relative importance of others × measurement score of subjective norm	0.03	0.27 **	0.27 **	0.66 **	0.14 **	0.15 *
Relative importance of self × measurement score of self-identity	0.11	0.47 **	0.42 **	0.70 **	0.15 **	0.22 **

** significant at 0.01; * significant at 0.05. Note: The number of cases varied due to missing data.

## Data Availability

Data sharing is not applicable to this article.

## Appendix A. Survey Items

Survey items for the intention of eating a low-fat diet in Study 1*

*Attitude toward behavior*

(1) “For me, eating a low-fat diet during the next week is” (“1 = unpleasant” and “7 = pleasant”);
(2) “For me, eating a low-fat diet during the next week is” (“1 = negative” and “7 = positive”);
(3) “For me, eating a low-fat diet during the next week is” (“1 = bad” and “7 = good”).

*Subjective norm*

(1) “People who are important to me would disapprove/approve of my eating a low-fat diet during the next week” (“1 = disapprove” and “7 = approve”);
(2) “People who are important to me think I should not/should eat a low-fat diet during the next week” (“1 = should not” and “7 = should”);
(3) “People who are important to me want me to eat a low-fat diet during the next week” (“1 = strongly disagree” and “7 = strongly agree”).

*Self-efficacy*

(1) “If you wanted to, how confident are you that you will be able to eat a low-fat diet during the next week?” (“1 = not very confident” and “7 = very confident”);
(2) “If you wanted to, to what extent do you see yourself as being capable of eating a low-fat diet during the next week?” (“1 = very incapable” and “7 = very capable”);
(3) “If I wanted to, I believe I am able to eat a low-fat diet during the next week” (“1 = definitely do not” and “7 = definitely do”).

*Self-identity*

(1) “I think of myself as a healthy eater” (“1 = strongly disagree” and “7 = strongly agree”);
(2) “I think of myself as someone who is concerned with healthy eating” (“1 = strongly disagree” and “7 = strongly agree”);
(3) “I think of myself as someone who is concerned with health consequences of what I eat. (“1 = strongly disagree” and “7 = strongly agree”).

*Intention*

(1) “I intend to eat a low-fat diet during the next week” (“1 = definitely do not” and “7 = definitely do”);
(2) “I plan to eat a low-fat diet during the next week” (“1 = definitely do not” and “7 = definitely do”);
(3) “I want to eat a low-fat diet during the next week” (“1 = definitely do not” and “7 = definitely do”).

*Relative importance of others vs. self*

“Please allocate 10 points between the two sources below to indicate the extent of their impact on your decision to eat a low-fat during the next week. Please use whole numbers.
People who are important to you____; Yourself____”

Note: * Survey items for the other intentions in both studies are similar to the items used for the intention of eating a low-fat diet, except for the context of the intention.
